# Surgical Management of Emphysematous Gastritis in a Postpartum Female: A Case Report and Literature Review

**DOI:** 10.7759/cureus.31595

**Published:** 2022-11-16

**Authors:** Fawwaz Almajali, Lauren Farley, Julia Sakach, Alexandra J Phocas, Matthew Pieper

**Affiliations:** 1 General Surgery, Saint Louis University School of Medicine, Saint Louis, USA; 2 Trauma, Saint Louis University School of Medicine, Saint Louis, USA

**Keywords:** gastrectomy, dilated stomach, acute care surgery and trauma, gastric body necrosis, gastritis, gastric emphysema, emphysematous gastritis

## Abstract

Emphysematous gastritis is a form of gastritis characterized by both gastric inflammation and the presence of intramural gas. Its occurrence is rare, and its presentation is non-specific. Consequently, no definitive guidelines for management have been outlined. We herein detail the diagnosis and surgical management of a female with complicated emphysematous gastritis following a cesarean section delivery. In light of the gastric ischemia noted on esophagogastroduodenoscopy, the decision was made to proceed with surgical management to ascertain the extent of necrosis. Following a partial gastrectomy, the patient had an uneventful postoperative course and met feeding milestones. Given the high morbidity rate of emphysematous gastritis and the success of our intervention, we propose the utilization of exploratory laparoscopy/laparotomy in patients with identified or highly suspected gastric ischemia. This aids in the characterization of ischemia and guides decision-making on the extent of gastric resection (partial versus complete gastrectomy).

## Introduction

Emphysematous gastritis is a rare form of gastritis with a mortality rate of more than 50% [1]. It is characterized by gastric inflammation with intramural gas [2]. It was first described in 1889 by Frankel [3], and since then, fewer than 100 cases have been reported in English literature [4]. It has a non-specific presentation, and the diagnosis is established radiologically in patients with predisposing risk factors. It is crucial to distinguish emphysematous gastritis from gastric emphysema, a relatively benign condition that usually occurs in secondary gastric mucosal trauma [5]. No clear guidelines are available for emphysematous gastritis management; however, medical treatment and surgery, when appropriate, improve the survival rate. Here, we present the management of complicated emphysematous gastritis with gastric necrosis following an uncomplicated cesarean section delivery in a 39-year-old female with known type 2 diabetes mellitus.

## Case presentation

A 39-year-old G3P2102 with a history of type 2 diabetes mellitus, noncompliance with medication, hypertension, and depression was admitted for hyperglycemic control and superimposed preeclampsia with severe features at 33 weeks and three days gestational age. She remained admitted until induction of labor at 34 weeks gestation. A primary cesarean section was pursued due to the maternal inability to accommodate lithotomy positioning for vaginal delivery. A chart review showed multiple presentations to the emergency department four to five months prior to admission with complaints of nausea, emesis, and abdominal pain that were attributed to pregnancy at that time.

**Figure 1:**
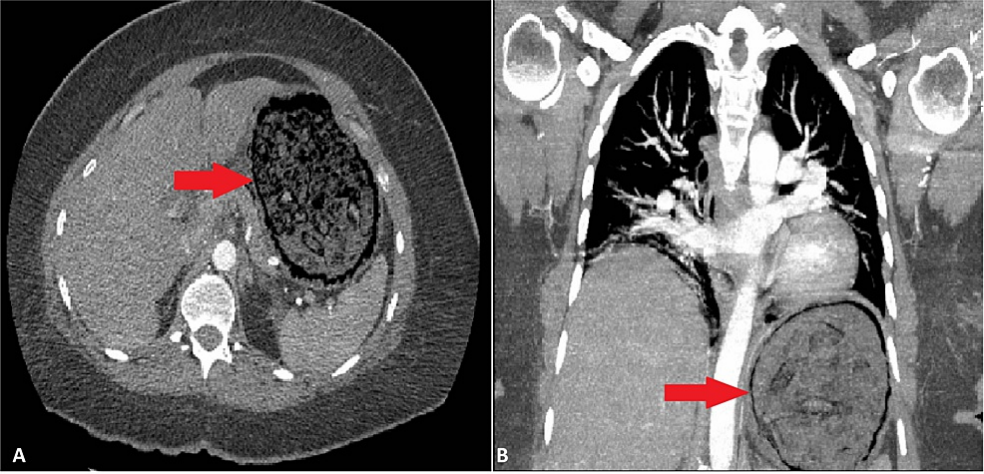
CT scan demonstrating air in the gastric wall (red arrow) on axial (A) and coronal (B) views.

On postpartum day 1, she reported abdominal pain with nausea and emesis that was treated with antacids and antiemetics. However, her symptoms persisted the following day, and she was noted to be tachycardic (120-130) but afebrile. Laboratory workup was significant for a new leukocytosis (16.4 × 10^3^/µL) with a left shift (88.5% neutrophils). A chest computerized tomography (CT) angiogram was ordered for concern of a pulmonary embolism which revealed evidence of emphysematous gastritis with air in the gastric wall and no evidence of pulmonary embolism (Figure 1).

**Figure 2:**
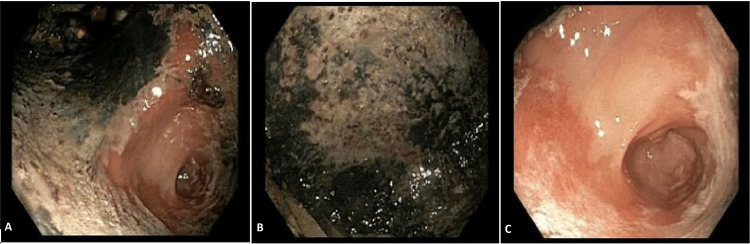
Esophagogastroduodenoscopy demonstrating necrotic and ulcerated mucosa in the fundus (A) and gastric body (B,C).

The gastroenterology team was consulted and recommended starting ampicillin/sulbactam, pantoprazole, and performing an esophagogastroduodenoscopy the next day that showed necrotic, erythematous, friable, and ulcerated mucosa in the cardia, gastric fundus, and gastric body without evidence of perforation (Figure 2). A nasogastric tube was placed under direct visualization.

General surgery was consulted and recommended starting piperacillin/tazobactam and micafungin. Repeat CT abdomen/pelvis revealed no progression of emphysematous gastritis or perforation. The patient was transferred to our hospital for a higher level of care.

**Figure 3:**
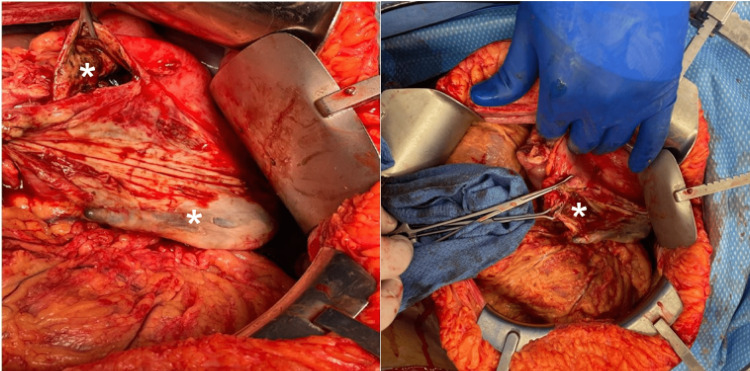
Intraoperative findings of transmural necrosis of the fundus and gastric body (asterisk) with visible nasogastric tube.

Upon arrival, she was still reporting abdominal pain and nausea. She was afebrile and tachycardic at 120-130. Laboratory workup was significant for leukocytosis (17.3 × 10^3^/µL) without lactic acidosis. She was started on piperacillin/tazobactam, and fluconazole. Due to concerns of full-thickness necrosis secondary to gastroparesis, given her history of uncontrolled diabetes, she underwent diagnostic laparoscopy with esophagogastroduodenoscopy, which confirmed full-thickness necrosis of the anterior wall of the gastric fundus and part of the body. The decision was made to convert to exploratory laparotomy, and a 15 cm ×15 cm resection of the gastric fundus and body was performed (Figure 3).

The gastrostomy was closed using 2-0 vicryl in a continuous locking fashion, followed by 2-0 silk in an interrupted Lembert fashion. The abdomen was left open, and a wound vacuum was placed with the plan to return to the operating room after 48 hours for reevaluation. On postoperative day 2, she underwent a re-exploratory laparotomy that showed an intact suture line, and intraoperative esophagogastroduodenoscopy showed persistent diffuse mucosal ischemia with a negative leak test; therefore, a Jackson-Pratt drain was placed near the suture line and a feeding jejunostomy tube was inserted distally. Histopathology showed complete mucosal necrosis with some areas of transmural necrosis and a negative immunostain for *Helicobacter pylori*.

Her postoperative course was uneventful. She was extubated the following day and started on a trickle feed through the jejunostomy tube that was advanced to goal as tolerated. She had a return of bowel function on postoperative day 6. Given the extent of mucosal ischemia, an abdominal CT scan with oral contrast was performed on postoperative day 8 and showed no leak; therefore, she was started on a clear liquid diet. An upper gastrointestinal series was performed and was consistent with delayed gastric emptying; hence, metoclopramide was started. A broad-spectrum antibiotic was continued for 14 days postoperatively due to the gross spillage of food content during the index operation. The patient was discharged on postoperative day 15 on a limited clear liquid diet (500 ml every 12 hours) and tube feedings through the jejunostomy tube.

On clinic follow-up one week after discharge, the patient was doing well, tolerating her clear liquid diet and tube feeds with no abdominal pain, nausea, or emesis; therefore, the diet was advanced to a full liquid/puree diet with the plan to repeat esophagogastroduodenoscopy in two weeks.

## Discussion

Emphysematous gastritis was first described by Frankel in 1889 [3]. The exact etiology remains unclear, as the stomach has a protective mucosal layer, an acidic pH, and a rich blood supply that provide adequate oxygenation, tissue repair, and resistance to infection [6]. Many risk factors have been identified, including corrosive ingestion, alcohol, gastroenteritis, gastric surgery, chronic use of nonsteroidal anti-inflammatory drugs (NSAIDs) and steroids, chronic obstructive pulmonary disease (COPD), diabetes, and immunosuppression [7]. Common causative microorganisms are *Staphylococcus aureus*, *Streptococci, E. coli*, Clostridium species, Enterobacter species, Pseudomonas, Proteus species, and Candida [8].

An important distinguishing factor in our report is that the patient demonstrated symptoms of diabetic gastroparesis based on her multiple presentations to the emergency department with emesis and abdominal pain (attributed to pregnancy at that time), which also predisposed her to develop emphysematous gastritis. In the event of gastroparesis and gastric dilation, the intraluminal pressure can exceed the gastric venous pressure, usually greater than 20 mmHg, impairing intramural blood flow and leading to gastric wall ischemia and necrosis [9,10].

Emphysematous gastritis has a non-specific presentation, including nausea, vomiting, abdominal pain, abdominal tenderness on examination, and leukocytosis. A CT scan is more sensitive and specific than abdominal films, with a classical finding of gastric wall thickening and air steaks or pockets within the gastric wall [7,11]. In addition, portal vein gas can be seen and is associated with a higher mortality rate [12].

It is crucial to distinguish emphysematous gastritis from gastric emphysema due to their overlapping radiological findings. Gastric emphysema results from mechanical injury to the stomach mucosa, either from a direct injury (nasogastric tube or esophagogastroduodenoscopy) or from increased intraluminal pressure within the stomach, i.e., barotrauma, leading to the introduction of air into the stomach wall [5,11]. As a benign, asymptomatic condition, gastric emphysema often resolves spontaneously unless complications occur. However, in emphysematous gastritis, there is a septic source in the gastric wall that can lead to an acute abdomen and require treatment [5]. Therefore, the differentiation between emphysematous gastritis and gastric emphysema should rely on clinical presentation, predisposing factors, and blood tests in addition to radiological findings.

The utilization of esophagogastroduodenoscopy remains controversial. While some experts emphasize urgent esophagogastroduodenoscopy to rule out gastric ischemia and the need for laparotomy [5,7]. Others recommend avoiding esophagogastroduodenoscopy due to the risk of perforation [13], although esophagogastroduodenoscopy has been proven safe with a very low risk for gastric perforation and has been utilized to guide treatment and assess recovery [3,14].

In this report, we present the case of a 39-year-old postpartum female with postpartum emphysematous gastritis and gastric necrosis requiring surgical intervention. While there are many points of discussion, in this case the two most relevance from a surgical perspective are the initial antibiotic choice and the need for operative intervention.

The literature review showed two management strategies, non-operative versus operative, based on the clinical stability and evidence of necrosis on esophagogastroduodenoscopy [5]. In our case, the decision to proceed surgically was made due to concerns for full-thickness necrosis after esophagogastroduodenoscopy and a worsening clinical picture after the initiation of medical management. Nasser et al. concluded that surgical exploration should be reserved for patients who failed non-operative management, became hemodynamically unstable, or developed peritonitis [15]. Upon review of past case reports, additional indications included concerns for sepsis and perforation [16,17]. In patients who underwent surgical management, procedures varied from partial gastrectomy to full gastrectomy with esophagojejunostomy, and outcomes ranged from complete recovery to death [16-20]. A 2017 review by Watson et al. found that surgical intervention did not confer significant mortality benefits when comparing outcomes from before and after the year 2000, and the only statistically significant predictor of mortality was the length of stay [7]. However, Watson et al. noted that before 2000, when 62.5% of patients underwent exploratory laparotomy, many were not initially managed medically, in contrast with after 2000, when only 22.2% of patients underwent exploratory laparotomy in conjunction with enhanced medical management and better surveillance through advances in biomedical technology [7]. Therefore, surgical benefits would likely be seen in populations that were refractory to conservative therapy and would otherwise continue to decompensate in patients such as this.

## Conclusions

Given the limited available data and the lack of a standardized clinical decision-making pathway for this rare condition, we propose the following strategy. When the diagnosis of emphysematous gastritis is confirmed by CT scan, immediate initiation of medical management is necessary, including nulla per os (NPO) status, a nasogastric tube, broad-spectrum antibiotics (e.g., piperacillin/tazobactam), and an antifungal agent if needed. Medical management should be followed by an esophagogastroduodenoscopy to assess for the presence of mucosal ischemia. In the absence of ischemia, we recommend continued non-operative management. However, if ischemia is present, we propose further investigation with an exploratory laparoscopy/laparotomy to further characterize the degree of ischemia and to determine whether a partial or complete gastrectomy is required.

By introducing this proposed management strategy, we aim to establish a more delineative, systematized pathway for treatment that eliminates ambiguity and optimizes patient outcomes.
